# Zinc as an adjunct treatment for reducing case fatality due to clinical severe infection in young infants: study protocol for a randomized controlled trial

**DOI:** 10.1186/s40360-017-0162-5

**Published:** 2017-07-10

**Authors:** Nitya Wadhwa, Sudha Basnet, Uma Chandra Mouli Natchu, Laxman P. Shrestha, Shinjini Bhatnagar, Halvor Sommerfelt, Tor A. Strand, the zinc sepsis study group, Siddarth Ramji, K. C. Aggarwal, Harish Chellani, Anuradha Govil, Mamta Jajoo, N. B. Mathur, Meenakshi Bhatt, Anup Mohta, Imran Ansari, Srijana Basnet, Ram H. Chapagain, Ganesh P. Shah, Binod M. Shrestha

**Affiliations:** 1 Pediatric Biology Centre, Translational Health Science and Technology Institute, Faridabad, Haryana India; 2 Department of Child Health, Institute of Medicine, Tribhuvan University, Kathmandu, Nepal; 3 Department of Neonatology, Maulana Azad Medical College and associated Lok Nayak Hospital, New Delhi, India; 4 Department of Pediatrics, Vardhman Mahavir Medical College & Safdarjung Hospital, New Delhi, India; 5 Department of Pediatrics, Kasturba Hospital, Delhi, India; 6 Department of Pediatric Medicine, Chacha Nehru Bal Chikitsalaya, Delhi, India; 7 Department of Pediatric Surgery, Chacha Nehru Bal Chikitsalaya, Delhi, India; 8 Department of Pediatrics, Patan Academy of Health Sciences, Lalitpur, Nepal; 9 Medical Department, Kanti Children’s Hospital, Kathmandu, Nepal; 10 Centre for Intervention Science in Maternal and Child Health, Centre for International Health, University of Bergen, Bergen, Norway; 11 Department of Research, Innlandet Hospital Trust, Lillehammer, Norway

**Keywords:** Sepsis, Severe infection, Infants, Neonate, Zinc, India, Nepal

## Abstract

**Background:**

An estimated 2.7 of the 5.9 million deaths in children under 5 years of age occur in the neonatal period. Severe infections contribute to almost a quarter of these deaths. Mortality due to severe infections in developing country settings is substantial despite antibiotic therapy. Effective interventions that can be added to standard therapy for severe infections are required to reduce case fatality.

**Methods/Design:**

This is a double-blind randomized placebo-controlled parallel group superiority trial to investigate the effect of zinc administered orally as an adjunct to standard therapy to infants aged 3 days up to 2 months (59 days) hospitalized with clinical severe infection, that will be undertaken in seven hospitals in Delhi, India and Kathmandu, Nepal. In a 1:1 ratio, we will randomly assign young infants to receive 10 mg of elemental zinc or placebo orally in addition to the standard therapy for a total of 14 days. The primary outcomes hospital case fatality, which is death due to any cause and at any time after enrolment while hospitalized for the illness episode, and extended case fatality, which encompasses the period until 12 weeks after enrolment.

**Discussion:**

A previous study showed a beneficial effect of zinc in reducing the risk of treatment failure, as well as a non-significant effect on case fatality. This study was not powered to detect an effect on case fatality, which this current study is. If the results are consistent with this earlier trial, we would have provided strong evidence for recommending zinc as an adjunct to standard therapy for clinical severe infection in young infants.

**Trial registration:**

Universal Trial Number: U1111-1187-6479, Clinical Trials Registry – India: CTRI/2017/02/007966: Registered on February 27, 2017.

## Background

It has been estimated that in 2015 nearly 2.7 of the 5.9 million deaths in children under 5 years of age occurred in the neonatal period [1] and almost ¾ in the first week of life [2] More than 70% of the neonatal deaths occur in Africa and South East Asia [3]. Severe infections like pneumonia and sepsis contribute to almost a quarter of these deaths [3, 4] and are also a major cause of hospitalization in infants [5]. Mortality due to severe infections in developing country settings is substantial despite antibiotic therapy [6]. Effective interventions that can be added to standard therapy for severe infections are required to improve clinical outcomes and case fatality.

In India, more than a quarter of the annual 1 million neonatal deaths can be ascribed to such severe infections [6]. Also in Nepal, sepsis is a leading cause of death in neonates, and the second most frequent reason for hospitalization [7]. While appropriate antibiotics are available in many hospitals in low and middle income countries, second-line antibiotics may be unavailable or are prohibitively expensive in peripheral health facilities. It is important to develop inexpensive, effective and accessible interventions that can be added to standard therapy for severe infections to improve treatment outcomes and reduce case fatality.

In a recent randomized placebo-controlled trial conducted in 3 tertiary hospitals in New Delhi, we found that 10 mg of elemental zinc given daily to 7 to 120 days old infants treated with antibiotics for probable serious bacterial infection (PSBI) carried a 40% (95% CI 10 to 60%) efficacy against treatment failure [8]. The absolute risk reduction was 6.8% (95% CI 1.5 to 12.0%), indicating that 15 (95% CI 8 to 67) infants would need to be treated with zinc in addition to antibiotics to prevent one treatment failure. We saw an even larger efficacy against treatment failure (54%; CI 20 to 74%) when analysis was restricted to 1 week to 2 month old infants, among whom only 11 (95% CI 6 to 37) would need to receive adjunct zinc treatment to prevent one treatment failure. This study of 700 infants is, to our knowledge, the first report of the efficacy of zinc in the treatment of PSBI in infants. However, the study was not powered to estimate the effect of zinc on case fatality. Thus, although the point estimate for the efficacy against death was the same as that against treatment failure, (43%; CI −23% to 73%), the study was not designed or powered to measure an effect on death and thereby not a strong driver for policy change.

Our study question is whether adjunct treatment with zinc will reduce the risk of death in infants with clinical severe infection. It is based on the promising findings of the above-mentioned trial and on the knowledge that zinc is crucial for immune function [9–11]. It is our hope that our large, multicentre study powered to examine the effect of zinc on case fatality from clinical severe infection would contribute critical evidence towards revising treatment recommendations for low resource settings in South Asia and elsewhere.

The aim of this double-blind randomized placebo controlled trial is to measure the efficacy of zinc administered orally as an adjunct to standard therapy to infants aged 3 days to 2 months hospitalized with clinical severe infection on reducing case fatality during hospitalization. We also propose to estimate the efficacy of zinc on reducing the extended case fatality risk, i.e. the risk of death until 12 weeks from the day of enrolment.

## Methods/design

### Study design and aims

Our hypothesis is that 10 mg of elemental zinc administered orally as an adjunct to standard therapy to infants aged 3 days up to 2 months (59 days) hospitalized with clinical severe infection identified using an adaptation of the WHO Integrated Management of Childhood Illnesses (IMCI) criteria will bring about a relative case fatality risk reduction of at least 30%, both during hospitalization for the illness in question as well as for the period up to 12 weeks from the day of enrolment.

Death being our primary outcome, the secondary outcome measures will be (i) failure of primary treatment, defined as a need to change antibiotics OR requirement for life support OR death, (ii) time to cessation of clinical signs, (iii) time to discharge, and (iv) death or severe illness at any time after discharge from hospital until 12 weeks from day of enrolment. The trial will also include mechanistic studies of the immune system during the illness episode.

### Study setting

**Fig. 1:**
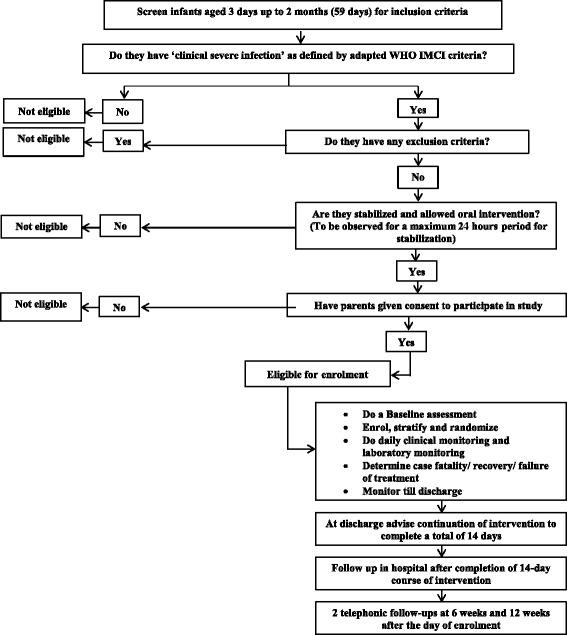
Flow diagram of the trial

The flow of the participants through the study is shown in the Fig. 1. We will recruit infants into the trial in 7 hospitals: 4 in Delhi, India, Maulana Azad Medical College (MAMC) and associated Lok Nayak Hospital, Vardhman Mahavir Medical College & Safdarjung Hospital (VMMC & SJH), Chacha Nehru Bal Chikitsalaya (CNBC) and Kasturba Hospital (KH); and 3 in the Kathmandu valley, Nepal, Patan Hospital [PH], Kanti Children’s Hospital (KCH), and Institute of Medicine (IOM). These are secondary level hospitals with similar standard of care, which will simplify standardization of Standard Operating Procedures (SOPs). Each of the seven hospitals has a large patient load, including of young infants with clinical severe infection. The overall implementation of the study will be coordinated by the Pediatric Biology Centre (PBC) at the Translational Health Science and Technology Institute (THSTI), in Faridabad, India; PBC will also directly oversee the work in the 4 Indian hospitals and will coordinate the immunobiological studies. The implementation of the study at the Nepal hospital sites will be coordinated by IOM. The Centre for International Health (CIH) at the University of Bergen and Innlandet Hospital Trust will work closely with THSTI, and IOM both in the overall coordination as well as in the immunobiological studies.

### Eligibility

We have adapted the inclusion criteria from the WHO IMCI [12] and Integrated Management of Neonatal and Childhood Illnesses (IMNCI) strategy [13] to identify sick infants with clinical severe infection [14]. Infants 3 days to 2 months of age attending the out patients department (OPD) or emergency services of the hospitals will be screened by a study nurse/medical officer and will be eligible for enrolment if any one or more of these five signs are present: hypothermia (axillary temperature < 35.5 °C), movement only when stimulated, stopped feeding well, severe chest indrawing and fever (axillary temperature ≥ 38.0 °C). To avoid including neonates in whom birth asphyxia is the reason for his or her signs, a criterion for inclusion will be that the infant should have been well at some point from birth till the current illness episode. The infants fulfilling the inclusion criteria will be excluded if they have the following conditions: weight-for-age < −4.5z at presentation, infants with surgical or other conditions that interfere with oral/nasogastric administration of the intervention (zinc sulphate or placebo tablets), infants requiring exchange transfusion, those who have received zinc in the last 48 h and those who have received injectable antibiotics for 48 h or more before presentation to hospital. Those infants who fulfill the criteria for clinical severe infection and do not have any exclusion criteria but are very sick and not allowed oral/nasogastric intervention will not be enrolled immediately but will be observed by the study nurse/medical officer during a ‘stabilization period’ for a maximum period of 24 h. Infants who after stabilization continue to have at least one sign of ‘clinical severe infection’ and no exclusion criteria and thereby fulfill eligibility criteria will be approached, i.e. their parents/guardians will be requested to give their written informed consent for their infant’s participation in the study.

### Approvals

The study protocol was assessed by the GLOBVAC program of the Research Council of Norway, before it allotted funding for the trial, and three independent expert reviewers assessed the protocol before The Centre for Intervention Science in Maternal and Child Health (www.cismac.org) provided its grant. Ethics clearance has been obtained from all the seven hospitals, THSTI Institutional Ethics Committee (IEC), Nepal Health and Research Council (NHRC), and Regional Committee for Medical and Health Research Ethics (REK) in Norway. Parents/guardians of infants eligible for the trial will be asked for written informed consent or thumbprint from those who are illiterate (in the presence of an impartial witness), declaring their willingness to have their infant participate in the trial. The participant information sheet will be in the local language and will detail the focus of the study along with the associated risks and benefits for the infant.

### Randomization, allocation concealment and masking

Eligible infants enrolled in the trial will be randomly assigned to receive zinc sulphate or placebo as tablets dispersed in breast milk or for those infants who are not breast fed in clean water. They will be stratified by hospital and by the presence or absence of diarrhea. A statistician/scientist not otherwise involved in the trial generated the randomization sequences using STATA (StataCorp, College Station, Texas, USA) such that, within each hospital and stratum, infants are randomized to receive zinc or placebo in a 1:1 ratio in permuted blocks of 8. A copy of the generated sequence is kept with the statistician/scientist and duplicate copies will be kept safely with another scientist not involved in the study and will not be available to any of the investigators until all data has been collected and cleaned, and the database is locked.

Identically looking blister packs containing dispersible tablets with or without zinc sulphate have been procured. The blister packs have been sequentially labeled by a team not involved in the trial with a unique serial number according to the generated randomization sequence.

The study nurse or medical officer will sequentially assign the unique enrolment ID to the enrolled infant. This assigned enrolment ID will be the same as the unique serial number on zip-lock bag containing 4 blister packs of 10 dispersible tablets each, assigned to that enrolled infant.

The packing and physical appearance of the zinc and placebo dispersible tablets are identical. In addition, the zinc and placebo tablets are similar in taste and appearance. This will ensure that the participant, investigator as well as the study team administering the intervention and ascertaining the outcome are unaware of group allocation. Masking will be maintained during data analysis by coding the treatment allocation with two letters.

### Intervention

Zinc (each tablet containing 5 mg of elemental zinc) and placebo are being manufactured by a GMP certified company L.P. Rodael, France and procured from Nutriset, France (www.nutriset.fr).

Once eligibility has been determined, the study nurse/medical officer will take a zip-lock bag labelled with the infant’s unique enrolment ID and assign it to the infant and give 5 mg of the intervention dissolved in 2.5 mL fluid (expressed breast milk or clean water) orally to the infant. Thereafter, the study nurse/medical officer will be responsible for giving one tablet of the assigned intervention to the infant every 12-h till hospital discharge. The infant will be observed for 30 min after each dose, which will be re-administered if he or she vomits within the observation period. A maximum of two attempts will be made to administer each dose. If an infant is unable to tolerate two consecutive doses due to vomiting, that dose will be skipped, following which the next and subsequent doses will be given to the enrolled infant. A careful log of successful and unsuccessful tablet administrations will be kept.

In those instances where an enrolled infant is not allowed oral feeds, the suspension will be given using a nasogastric tube as will the required feeds. In the few cases where the enrolled infant develops necrotising enterocolitis or tense abdominal distension or any such condition where the treating physician decides not to allow the tablets​ to be given, they will be withheld until there is clinical improvement. While all infants will be included in the main, intention-to-treat, analysis, those who receive 50% or more of the projected doses in the first 5 days after enrolment will be included in the per protocol analysis.

At discharge, the parent(s)/caregiver(s) will be advised to continue giving the dispersible tablets twice daily to complete a total of 14 days of treatment. The caregiver will be trained and advised to follow the same protocol of administering the tablets dispersed in liquid as was being followed during the hospital admission and will be asked to repeat the intervention once if their baby vomits within 30 min of administration. The caregiver will be equipped with a small durable booklet to record every successful administration as well as any failed attempts to administer the intervention.

### Co-interventions

The treatment of enrolled infants of ‘clinical severe infection’ with standard antibiotics and other medications will be given based on our SOPs, which have been standardized across the seven hospitals. The antibiotics received by the enrolled participant will be documented in the case report forms along with the route, dose and frequency.

All other therapy prescribed to the enrolled infant by the treating physician like supplemental oxygen, intravenous fluids, multivitamins, etc. will also be documented in the case report forms along with the route, dose and frequency. Care will be taken that the enrolled infant is not prescribed zinc inadvertently, particularly in multivitamin drops or creams for local application for perianal rash.

### Clinical data collection

Study nurses/medical officers will examine the enrolled infants for all clinical features of clinical severe infection every 6 h, or more often if indicated, until discharge from the hospital. They will record nude weights at enrolment and every 24 h till discharge. The research officers (pediatricians) will oversee at least one daily monitoring conducted by each study nurse/medical officer. The decisions for all outcome measures will be made by the research officer in consultation with an experienced pediatrician (site investigator) based on the closely supervised 6 hourly monitoring done by the study nurse/medical officer.

One follow-up visit to the hospital will be scheduled after they have completed the 14-day course of intervention (around day 15 after enrolment) for which they will be asked to bring the zip-lock bag containing the 4 blister packs. Data on the infant’s health will be collected and a ‘residual pill count’ will be made to estimate the compliance.

After this follow up visit they will be contacted on telephone at one and a half months and again at 3 months from the day of enrolment. During the telephonic interview, information will be collected on the infant’s health, feeding, any severe illness requiring hospitalization or any other serious adverse event, using structured forms.

Parents/caregivers of infants will be encouraged to report any hospitalization within the 12-week study period after enrolment, to the study team through a visit to the hospital site or a call made to the study team.

### Standard case management

Each hospital site has a fixed antibiotic treatment algorithm and follows the protocol strictly. Recommended doses of intravenous ampicillin or amoxicillin-clavulanic acid and an aminoglycoside (amikacin or gentamicin) will be given when infants either have not received antibiotic treatment or have been given oral antibiotics for the current illness episode. A third generation cephalosporin (cefotaxime or ceftriaxone) and an aminoglycoside (amikacin or gentamicin) will be initiated in those infants that have already been treated with injectable antibiotics (for <48 h) prior to admission. In case of suspected meningitis, ceftriaxone or cefotaxime combined with amikacin will be started on admission. In case of suspected staphylococcal infection, cloxacillin or amoxicillin-clavulanic acid will be given with an aminoglycoside and in suspected staphylococcal meningitis vancomycin combined with an aminoglycoside will be administered. The duration of antimicrobial therapy will be 7–10 days, extendable to 3 weeks in meningitis. Intravenous fluids, temperature maintenance and oxygen will be provided as supportive therapy. The treating physicians who are also site investigators will be responsible for the medical treatment of the enrolled infants with clinical severe infection. The study staff will be responsible for clinical and safety monitoring.

### Laboratory procedures

#### Blood specimens

Study nurses/medical officers will collect blood specimens at baseline (enrolment), at 48–72 h of enrolment and at discharge. This will include 2 ml blood at enrolment, required for standard care, including blood culture by BACTEC and a septic screen which includes C-Reactive Protein (CRP), total leukocyte count, absolute neutrophil count, micro erythrocyte sedimentation rate and band cell to neutrophil (I:T) ratio. All investigations necessary for standard care will be done with techniques that use small volumes of blood at the study hospital or at an accredited laboratory in the close vicinity of the hospital.

The study technicians will separate the sera for zinc and soluble markers of inflammation at the hospital site, and then transport the samples in insulated boxes with ice-packs to the central laboratory at THSTI for the Indian hospitals, or IOM for the Nepali hospitals, where it will be stored at −70 °C until analysis. Serum concentrations of zinc will be measured in a subset of the enrolled infants. Serum CRP concentration will be measured using a commercial ELISA kit (Biocheck, Foster City, CA, USA) at the micronutrient lab at THSTI. The blood will be centrifuged at approximately 700 g for 10 min at room temperature.

#### Nasopharyngeal swab

Nasopharyngeal swabs will be collected at the time of enrolment for etiology and characterization of viral pathogens and transported to the central laboratory at THSTI/IOM at the earliest on ice (dry-ice if available) and will be stored in - 70 °C deep freezers till analysis. The stored swabs from the repository in Nepal will be transported to the central laboratory at THSTI in India twice a year.

### Outcomes

#### Primary outcomes

To estimate the efficacy of 10 mg elemental zinc administered orally as an adjunct to standard antibiotic therapy to infants aged 3 days up to 2 months (59 days) hospitalized with ‘clinical severe infection’ against

i. Case fatality. The case fatality risk is the proportion of children with ‘clinical severe infection’ who die due to any cause and at any time after enrolment while hospitalized for the illness episode.
ii. Extended case fatality. Time to death until 12 completed weeks from the day of enrolment.

#### Secondary outcomes

i. Failure of treatment. Defined as any one or more of the following events:
  - Death at any time during initial hospitalisation after enrolment, or
  - Requirement of life support defined as a need for ventilation or vasoactive drugs at any time after enrolment until hospital discharge, and/or
  - A change in antibiotics for one of the following circumstances:
  i) Persistence of signs that indicate ‘clinical severe infection’ present at the time of enrolment any time after 48 h of enrolment and prior to discharge.
  ii) Worsening of existing signs or appearance of new signs of ‘clinical severe infection’ any time after enrolment and prior to discharge.
  iii) Reappearance of signs of ‘clinical severe infection’ that the infant initially presented with at time of enrolment anytime after 48 h of disappearance and prior to discharge
ii. Cessation of signs of clinical severe infection: This will be defined as the beginning of a 48 h period with none of the signs of ‘clinical severe infection’.
iii. Death at any time after discharge from hospital until 12 completed weeks from the day of enrolment.
iv. Severe illness requiring hospitalisation at any time after discharge from hospital until the end of 12 weeks from the day of enrolment.
v. Immunobiological effects of zinc.

### Safety considerations, safety monitoring and adverse event reporting

Zinc has been shown to be safe in doses ranging from 5 to 45 mg per day when it has been used in both the treatment of acute diarrhea as short-term therapy and also as long-term supplementation in infants and young children in Asia, Latin America, and Africa [15–22]. All commonly used zinc salts (sulfate, acetate, and gluconate) have been found to be safe [21]. Infants with probable serious bacterial infection who were treated with zinc in our own earlier trial [8] did not demonstrate any adverse effects/events.

All adverse events (AE) and serious adverse events (SAE) will be recorded by the study nurse/medical officer. AEs are graded from 1 to 5 according to their severity according to Common Terminology Criteria for Adverse Events (CTCAE) [23]. All immediate adverse events following each dose of intervention and every 6 h follow-up, until discharge from hospital and till the infant completes the 14 days of the intervention like regurgitation or vomiting within 30 min of administration of first or subsequent dose of intervention, vomiting any time during the 14-day period, abdominal distension lasting >24 h any time during the period of intervention, appearance of new symptom, etc. will be documented for all infants.

SAE, that includes critical or life threatening illness or death, will be documented from the time of enrolment, throughout the study period. The study nurse/medical officer will also make three follow-up contacts after discharge of the infant from hospital. During each contact the study staff will collect data to ascertain SAEs and illness requiring hospitalization. All SAEs of death, development of signs of critical illness which are life threatening and severe illness requiring hospitalization after discharge (but during the study period) will be reported to the Ethics Committees (EC), the Data and Safety Monitoring Board (DSMB) and the sponsors of the study within 24 h of awareness of the event followed by a final report within 10 days. SAE relatedness to administration of the dispersible tablet with zinc sulphate or placebo will be judged by the investigator/designee, the Ethics Committee and the DSMB who will have access to all relevant investigations, clinical assessments and management details**.** All adverse events (AE) will be also be reported to the ECs, DSMB and sponsors at periodic pre-defined intervals.

### Follow up after adverse events

All adverse events will be followed up till resolution or stabilization as judged by the treating pediatrician (site investigator) and the principal investigator. All SAEs will be followed up until satisfactory resolution or until the treating pediatrician and the principal investigator deem the event to be chronic or the participant to be stable.

### Data and Safety Monitoring Boards (DSMBs)

There will one DSMB for the Indian and one for the Nepal hospital sites. The DSMB for the Indian hospital sites will comprise of a pediatrician, epidemiologist and a biostatistician. The DSMB for the Nepal hospital sites will comprise of three members including a statistician and a pediatrician. We will attempt to have one of the DSMB members overlapping in both the Indian and the Nepali DSMB. These DSMBs will be independent from the sponsor and will have no competing interests. The DSMBs will prepare a charter and decide a priori on study stopping rules, and will review SAE and AE reported in the study periodically. They will examine all infant deaths and other SAEs to decide if the study should be continued, based on the pre-decided stopping rules. After one third of the study participants have been enrolled and completed follow-up, the DSMBs will review the data and make recommendations concerning continuation, modification or termination of the study due to unexpectedly large beneficial effects or serious side effects and can suggest extension of the trial should the primary outcomes occur less often than anticipated.

### Quality control and quality assurance

Misclassification will be minimized by employing strict definitions of the outcomes, developing clear and concise SOPs, initial training before study initiation and repeated training during study implementation.

The supervisors of the study team who are medical doctors will ensure quality control through regular checks on all activities being performed in the study, such as screening of infants aged 3–59 days coming to the hospital OPD or emergency department, taking written consent from parents/guardians, enrolment of eligible infants, administering the intervention/placebo at enrolment and then every 12 hourly until discharge and advising the parents/caregiver to continue with the intervention at home after discharge in the same dose to complete a total of 14 days, follow-up of enrolled infants every 6 hourly during hospitalization, assessing for and documenting outcomes, reporting and management of serious adverse events, collection, immediate processing and storage of blood samples at the predefined time points, storage of the zinc and placebo tablets, calibration of clinical and laboratory equipment like infant weighing scale, infantometer, incubator, centrifuge, refrigerator, deep freezers, pipettes, etc.

Initial training and then repeated training during study implementation will be provided to the study staff on each relevant study activity. In order to ensure that the standardized protocol is followed at all sites and by all the study staff, standardization exercises will be done within each site and then between different sites at regular defined intervals.

Quality control (QC) of lab samples will be done using a standard protocol.

### Independent study audit

An independent auditor will be responsible for auditing the study. The auditing plan will include predefined periodic visits to the recruitment sites by the independent CISMAC-funded auditor(s) who will review relevant aspects of the study using standardized formats. The conclusions/recommendations arising from these visits will be communicated to the PIs and site investigators for corrective and preventive action (CAPA).

### Sample size and statistical plan of analysis

Based on the most recent annual audits undertaken in the district hospitals in India, the case fatality risk in infants <2 months with clinical severe infection is expected to be 10%. Assuming a 10% loss to follow-up, we will need to recruit 4140 infants (2070 in each group) to identify a clinically important (≥30%) relative risk reduction of death by administering zinc, with 90% power and 95% confidence. We assume that the power to detect differences in case fatality until the end of 12 weeks after enrolment is higher mainly because there will be more events over the longer follow up period.

### Plan of analysis

Using STATA, we will estimate the proportion of children who die in the two study (zinc and placebo) arms and the relative risks (RRs), risk differences (RDs) and its reciprocal, the numbers needed to treat (NNTs). Relevant baseline characteristics will be displayed by study arm. In case any of the baseline variables are unevenly distributed between the two study arms, and we suspect that this can confound our effect estimates, we will adjust for these baseline imbalances. For these analyses, we will be using generalized linear models of the binomial family with log and identity links, respectively. We will use the same methods to estimate the effect on deaths over the 3-month period. In addition, the differences between the groups will also be estimated using Cox proportional hazards models also taking time under observation into account.

#### Secondary outcomes

Likewise, we will compare the risks in the intervention and placebo arms of the trial, for the secondary outcomes.

For the secondary outcome of treatment failure, we will compare the risk in the intervention and placebo arms of the trial using

i. Composite definition of treatment failure (death and/or requirement of life support and/or need for change in antibiotics)
ii. Cause specific treatment failure.

The cause of treatment failure will be assigned by the worst outcome and the first cause of failure.

These secondary outcomes will be analyzed the same way as the primary outcomes. If an outcome-event can occur more than once, we will adjust for the repeated observations in statistical models. The secondary outcome of severe illness requiring hospitalization will also be analyzed in Poisson or negative binomial regression models (allowing for more than one event per child).

Time-to-event analyses will be done for the following primary and secondary outcomes:

**Time to death during hospitalization:** The event will be death at any time after enrolment while hospitalized for the illness episode. Infants where the caretaker withdraws consent for continuation in the study or for other reasons cannot be followed until recovery and discharge will be censored. We will also use Cox proportional hazards models to compare the time to death during initial hospitalization between study arms and will calculate the relative hazard of death during hospitalization between the intervention groups in (i.e. comparing time until death during hospitalization).

**Time to death until end of study period:** defined as time to death at any time after enrolment until 12 completed weeks thereafter. This analysis will consider the event as death at any time during the study period. Infants where the parent/guardian withdraws consent for continuation in the study or for other reasons cannot be followed until 12 weeks after enrolment in the study will be censored at the time point that they were lost to follow-up. We will use a Cox proportional hazards model and calculate the hazard ratios (HR).

**Time to cessation of signs of clinical severe infection**: The event will be cessation of signs of clinical severe infection. Infants, where the caretaker withdraws consent for continuation or for other reasons cannot be followed further, will be censored. Irrespective of whether treatment failure has occurred, we will use a Cox proportional hazards model to compare the time to cessation of signs of clinical severe infection between study arms.

**Time to failure of treatment**: This analysis will consider the event as treatment failure. Censoring infants who withdraw consent to continue in the trial we will use a Cox proportional hazards model to compare the time to treatment failure between study arms.

**Time to discharge:** This analysis will consider the event as discharge. Censoring infants who withdraw consent for continuation in the trial, we will use a Cox proportional hazards model to compare the time to discharge between study arms.

Like antibiotics, zinc may not exert an effect before 24 h of administration. All of the above analyses will therefore be repeated where the outcomes are redefined to occur only when the event takes place after 24 h of administering the first dose of zinc.

### Analyses of laboratory parameters

Serum zinc concentration and immunobiological readouts at discharge and the mean change from baseline to the second blood sample will be compared between the two groups using appropriate statistical test adjusting for relevant baseline imbalances when necessary.

### Subgroup analyses

We will estimate the efficacy of zinc in various subgroups based on (i) presence or absence of diarrhea on admission [8], (ii) hospital, (iii) age (<7 vs ≥7 days) (iv) presence of sepsis, either ‘culture positive’ (true pathogen detected in blood culture) or ‘clinical sepsis’ defined by a negative blood culture but a positive septic screen which is signalled by the presence of any two of the following parameters: total leucocyte count <5000/cmm; absolute neutrophil count <1500/cmm; band cell:neutrophil ratio > 0.2; micro ESR >15 mm at 1st hour and C-reactive protein levels >1 mg/dl (adapted from Center for Disease Control and Prevention criteria) [24]. In addition to displaying the RRs for each of the above-mentioned strata, we will also consider estimating heterogeneity of RRs and RDs using interaction terms in our regression models. Interaction analysis will also be conducted on an additive scale, according to Rothman [25] and Anderson [26] et al.

### Intention-to-treat (ITT) analysis

Our primary analysis will use an intention-to-treat approach where all infants assigned into the zinc or placebo group and whose outcome is known will be analyzed. In addition to a standard per protocol analysis, we will use Instrumental Variable Analysis (IVA) in an attempt to estimate the true effect of adjunct treatment with zinc had it been given to all children in the scheduled doses and intervals. IVA can also take into account the theoretical situation that babies allocated to the placebo arm receives zinc. The random allocation will be the instrument in these analyses. For per protocol analysis, infants who receive less than 50% of the projected doses in the first 5 days after enrolment will not be included in the analyses, well acknowledging that the ensuing effect estimates may not only be biased but will certainly represent an effect higher than what can be achieved even in our well-resourced secondary hospitals.

### Data management

The Data Management Centre (DMC) at THSTI will manage the study data.

#### Data collection, including type, format, scale and standards for data

The data will be collected on paper case report forms (CRFs) or electronically in an inhouse programmed SQL data-base capture system at the seven hospitals by the study nurses/medical officers supervised by the study physician. The forms will be developed by the DMC and will consist of details of all the variables with standard definitions and labels. Consistency and range checks will be inbuilt into the data entry screens such that these checks are applied during entry itself. Each form once entered by the data entry operator,/study nurse or medical officer will be transferred to the server at the DMC. The DMC will generate query forms which will be returned to the hospital research staff for concurrent verification.

#### Data security and storage

At least one backup of the data will be kept in the server at the DMC protected by a specific password and accessible to only authorized users. An additional backup of the data will be kept in a password protected external hard drive at a site away from the DMC at the coordinating centre. The DMC will send the data at predefined intervals to the central data repository at THSTI.

#### Data tracking, cleaning, and quality checks

The DMC will be responsible for initial cleaning of the data. Interim tabulations and scatter plots for some variables will be made at regular intervals to identify data errors. Special checks will be made on observations that are more than two or three standard deviations from the mean. There will be a regular feedback of errors from the DMC to the clinical sites for correction within 48 h.

### Record retention and archival

All the study documents including participant’s source data and documents will be archived by the study sites after the completion of the study, till the time the sponsor informs in writing to the study sites that they no longer need to maintain the study documents.

## Discussion

Our previous study showed a beneficial effect of zinc in reducing the risk of treatment failure, as well as a non-significant effect on case fatality among infants 1 week to 4 months of age with PSBI. This trial was not powered to detect an effect on case fatality [8]. If the results of the current trial are consistent with those of our earlier trial [8], we would provide strong evidence for recommending zinc as an adjunct to standard therapy for clinical severe infection in young infants. Further, as we propose to identify young infants with clinical severe infection using an adaptation of the WHO IMCI criteria, it would be easier to justify introduction of zinc in the national programs where IMCI/IMNCI is followed. We will also engage with policy-makers and program managers, at the state and at the national level and with WHO to suggest a consideration of introducing this simple, inexpensive medication as recommendations and guidelines following the process established by the Guideline Review Committee (GRC) of the WHO.

### Trial status

The first child was enrolled in March, 2017.

## Abbreviations

AE: Adverse event
CHN-CIH: Centre for International Health, University of Bergen
CHP/PCOR: Centres for Health Policy/Primary Care and Outcomes Research
CNBC: Chacha Nehru Bal Chikitsalaya
CRP: C-Reactive Protein
CTCAE: Common terminology criteria for adverse events
DMC: Data Management Centre
DNA: Deoxy ribonucleic acid
DSMB: Data and Safety Monitoring Board
ICU: Intensive care unit
IEC: Institutional Ethics Committee
IMCI: Integrated Management of Childhood Illnesses
IMNCI: Integrated Management of Neonatal and Childhood Illnesses
IOM: Institute of Medicine
KCH: Kanti Children’s Hospital
KH: Kasturba Hospital
MAMC: Maulana Azad Medical College
NHRC: Nepal Health and Research Council
OPD: Out patient department
PBC: Pediatric Biology Centre
PBMC: Peripheral blood mononuclear cell
PH: Patan Hospital
PSBI: Probable serious bacterial infection
REC: Regional Committee for Medical and Health Research Ethics
RNA: Ribonucleic acid
SAE: Serious adverse event
SJH: Safdarjung Hospital
SOP: Standard operating procedure
THSTI: Translational Health Science and Technology Institute
TU: Tribhuvan Univresity
VMMC: Vardhman Mahavir Medical College
